# Adapting the Hospitalist Work Style to Japan’s Work Style Reform: A Qualitative Study from the Perspectives of Japan-Trained Hospitalists in the United States

**DOI:** 10.31662/jmaj.2025-0512

**Published:** 2026-04-03

**Authors:** Michito Sadohara, Kunihiko Matsui

**Affiliations:** 1 Department of General Medicine and Primary Care, Kumamoto University Hospital, Kumamoto, Japan

**Keywords:** hospitalist, work-style reform, work-life balance, shift-based practice, physician well-being, structured handoff, task shifting, organizational change

## Abstract

**Introduction:**

In Japan, the Work Style Reform Bill, enacted in 2018, aimed to reduce excessive working hours and promote diverse work styles. Following the full enforcement of physician work-hour limits in 2024, attention has shifted toward developing sustainable work styles that maintain the quality of care while improving efficiency and physician well-being. The United States (U.S.) hospitalist model―characterized by shift-based inpatient practice―may offer relevant insights; however, its feasibility in Japan remains unclear. This study explored the feasibility, benefits, and challenges of adapting the hospitalist work style to Japan from the perspectives of Japan-trained hospitalists practicing in the U.S.

**Methods:**

Semi-structured interviews were conducted with three Japan-trained physicians currently working as hospitalists in the United States. Interviews examined cross-national system differences, the contribution of hospitalist practice to Japan’s reform goals, barriers to implementation, and recommendations for adaptation. Data were analyzed qualitatively using the Steps for Coding and Theorization method.

**Results:**

Four major themes emerged: (1) *systemic and structural differences between* Japan* and* the United States; (2) *the need to change traditional work culture*; (3) *practical strategies for implementation in Japan*; and (4) *public acceptance and readiness*. Participants emphasized that role differentiation, shift-based scheduling, and team-based care in the U.S. enable predictable workloads and promote physician well-being, contrasting with Japan’s continuous-duty culture and overlapping roles. Feasible strategies included structured scheduling, standardized handoffs, equitable workload distribution, task shifting, and pilot programs in large hospitals. Successful implementation was viewed as dependent on legal and institutional support and public acceptance grounded in demonstrable value.

**Conclusions:**

The hospitalist work style was well aligned with―and highly applicable to―Japan’s work style reform. Despite institutional differences, several components were considered immediately transferable. Sustainable integration, however, will require transformation of work culture, enhanced institutional and public understanding, and the accumulation of evidence to support broader implementation.

## Introduction

In Japan, long working hours among physicians have traditionally been customary and deeply embedded in the professional culture, limiting diverse work styles and undermining physician well-being. Persistent overwork has raised serious concerns regarding workforce sustainability, professional development, and the long-term quality of patient care. These challenges provided the critical impetus for national work style reform ^(1)^.

The Work Style Reform Bill, enacted in 2018, aimed to achieve three principal objectives: reducing overtime work, mitigating disparities between regular and non-regular employees, and promoting flexible work practices ^(2)^. During the five-year grace period for implementing work-hour limits for physicians ^(3)^, hospitals and physicians sought to establish sustainable and efficient workflows in compliance with the new regulations. Meanwhile, the Ministry of Health, Labour and Welfare (MHLW) encouraged voluntary institutional initiatives rather than mandating specific implementation strategies ^(4)^. Following full enforcement in April 2024, attention has shifted toward identifying physician work styles that can sustainably enhance both the quality of care and physician well-being.

The term *hospitalist*, first defined by Wachter and Goldman in 1996, refers to physicians who devote most of their time to the care of patients who are hospitalized, typically working under a shift-based schedule ^(5)^. The hospitalist model emerged in the United States (U.S.) in response to growing demands for efficient management of individuals with multiple chronic conditions and to reduce the increasing workload of primary care physicians traditionally responsible for inpatient care ^(6)^. Today, with more than 50,000 practitioners, hospitalists constitute the largest medical specialty group in the U.S., forming the core of inpatient medical services ^(7)^.

In contrast, the hospitalist model remains relatively new in Japan, where the term “hospitalist” has not been formally defined within policy frameworks. Unlike in the U.S., physicians working in Japanese hospitals typically provide both inpatient and outpatient care, which may contribute to role ambiguity, inefficiencies in patient management, and imbalances in workforce allocation ^(8)^. Consequently, the institutional compatibility, operational feasibility, and system-level implications of hospitalist practice in Japan remain insufficiently understood. To address this gap, examining the perspectives of physicians familiar with both Japanese and U.S. healthcare systems could provide valuable insights for adapting this model to Japan’s ongoing work style reforms.

By examining how Japan-trained physicians currently practicing as hospitalists in the U.S. perceive the feasibility, potential benefits, and challenges of introducing this work style into the Japanese healthcare system, this study elucidates key structural and cultural considerations relevant to Japan’s reform agenda. These insights are expected to inform future policy discussions, guide institutional initiatives, and support strategies that promote sustainable physician work styles and well-being.

## Materials and Methods

### Study design and participants

This qualitative study employed semi-structured interviews with Japan-trained physicians currently working as hospitalists in the U.S. These participants were purposively selected for their familiarity with both Japanese and U.S. healthcare systems, employment practices, and medical culture. Eligibility criteria were as follows: (1) prior clinical training in Japan, (2) current employment as a hospitalist in the U.S., and (3) provision of written informed consent to participate.

Given that physicians meeting these criteria were rare, purposive snowball sampling was used. In June 2018, we first contacted a hospitalist who had authored a Japanese book on hospital medicine and was affiliated with a U.S. teaching hospital with an established hospitalist program. This physician introduced an eligible colleague who agreed to participate and subsequently referred two additional participants. Recruitment occurred over approximately six months, and interviews were conducted in November 2018. Given the highly specific eligibility criteria and the exploratory qualitative design, the study prioritized conceptual depth over numerical representativeness. Sample size was determined pragmatically based on feasibility and participant rarity. Conceptual saturation was assessed iteratively; by the third interview, no substantively new concepts relevant to the research question emerged, and further recruitment was deemed unnecessary.

### Data collection and analysis

Approximately 30-minute semi-structured interviews were conducted in Japanese by Michito Sadohara with each participant individually in a private, quiet room at their workplace to ensure confidentiality.

Guided by the qualitative research question―“*How do Japan-trained hospitalists practicing in the United States perceive Japan’s work* *style reform?*”―the interviews explored experiences and perspectives relevant to informing Japan’s work style reform. The discussions focused on cross-national differences in cultural norms, institutional structures, and clinical work practices, as well as on elements readily applicable within the Japanese context.

The interview guide covered four domains: (1) systemic differences between Japanese and U.S. healthcare systems, (2) the potential contribution of hospitalist practice to work style reform, (3) barriers to implementation, and (4) recommendations for adaptation in Japan. All interviews were audio-recorded and transcribed verbatim. Initial automated transcription was performed using NVivo^Ⓡ^ (Lumivero Inc., Denver, CO, USA) and was subsequently reviewed and manually corrected for accuracy.

Data were analyzed using the Steps for Coding and Theorization (SCAT) method―an inductive qualitative analysis technique that offers a clear and systematic procedure for coding and theorization, even with small-scale interview datasets ^(9), (10)^. Following the four-step SCAT procedure, each interview transcript was segmented and coded as follows: (1) noteworthy words or phrases were extracted directly from the text; (2) paraphrases of these extracted expressions were generated; (3) external concepts accounting for the paraphrased expressions were identified; and (4) these concepts were synthesized into higher-order themes or constructs through contextual interpretation of the dataset. The four analytic steps were initially conducted by Michito Sadohara. Through iterative discussions between Michito Sadohara and Kunihiko Matsui, paraphrases and thematic assignments were refined to ensure interpretive consistency. The final set of themes and subthemes captured system-level, cultural, and operational factors relevant to the potential adaptation of hospital medicine in Japan.

### Ethical considerations

The study was approved by the Research Ethics Committee of Kumamoto University (Approval No. 1572). Written informed consent was obtained from all participants, and all data were anonymized during analysis.

## Results

### Participant characteristics

Three Japan-trained hospitalists practicing in the U.S. participated in the study (Table 1). All participants were male, and two were married. One physician had originally been affiliated with a university medical department and held a subspecialty qualification in Japan. Their experience as hospitalists ranged from four months to three years at the time of the interviews. One hospitalist reported that he had chosen the hospitalist career in the U.S. to achieve a better work-life balance.

**Table 1.** Participant Demographics and Professional Background.

	Hospitalist A	Hospitalist B	Hospitalist C
Gender/marital status	Male/married	Male/married	Male/not married
Nationality	Japanese	Japanese	Chinese
Interview duration	34 min, 54 sec	29 min, 57 sec	27 min, 54 sec
Clinical training and specialty in Japan	Institution-specific program at a private clinical training hospital in prefectural capital (pre-MHLW PGCT); general internal medicine	Institution-specific program at a university hospital in the national capital (pre-MHLW PGCT); general medicine rotation, respiratory medicine	Completed the mandatory two -year MHLW PGCT at a metropolitan clinical training hospital in the national capital
Prior clinical experiences in the U.S.	IM residency & fellowship; ABIM-certified; 4 years’ experience	IM residency & fellowship; ABIM-certified; 6 years’ experience	IM residency (U.S.)
Current position and years of hospitalist experience	Full-time hospitalist (attending), 1.5 years	Full-time hospitalist (attending), 3 years	Full-time hospitalist, 4 months
Motivation and opportunity to move to the U.S. and become a hospitalist	•Sought better work-life balance and family compatibility •Moved to the U.S. to pursue a hospitalist career	•Initially planned overseas research training •Chose U.S. clinical fellowship to continue clinical practice, leading to hospitalist work	•Learned about the hospitalist career during residency •Selected the role based on peer and senior influence

ABIM: American Board of Internal Medicine; IM: internal medicine; MHWL: Ministry of Health, Labour and Welfare; PGCT: postgraduate clinical training.

### Overview of emerging themes, storyline, and theoretical description

The analysis identified four major themes and several subthemes regarding the feasibility of adapting the hospitalist model to Japan (Table 2). The overarching storyline shared by all three participants, along with the corresponding theoretical interpretations, is summarized in Table 3. The Japan-trained hospitalists practicing in the U.S. emphasized that adapting the hospitalist model to Japan would require addressing systemic and cultural barriers rooted in differences in healthcare systems, employment structures, and physician roles. Key enabling factors included promoting work-life balance, establishing structured handoffs and equitable workload distribution, implementing task shifting, and initiating organizational reform through pilot programs in large hospitals. Successful implementation was viewed as dependent on public understanding and acceptance, legal flexibility for remote and delegated work, and broader cultural change toward team-based care.

**Table 2.** Themes and Subthemes.

1. Systemic and structural differences between Japan and the United States
1.1 Healthcare delivery and health insurance systems
1.2 Role differentiation between generalists and specialists
1.3 Employment structure and working conditions
2. Need to change traditional work culture
2.1 Incentives to work-life balance
2.2 Task shifting to other healthcare professionals
2.3 Overcoming cultural barriers and misconceptions
3. Practical strategies for implementation in Japan
3.1 Integration and reorganization of healthcare resources
3.2 Pilot program in large hospitals
3.3 Establishing structured handoffs and equitable workload
3.4 Remote access and digital flexibility
3.5 Delegation of authority and proxy orders
4. Public acceptance and readiness

**Table 3.** Description of Storyline and Theory Derived from Interviews with Three Hospitalists.

The storyline
The hospitalists, who had worked and practiced in both countries, recognized the challenges posed by the **systemic and structural differences between Japan and the United States** in physicians’ workstyle reform in Japan. These challenges were attributed to differences in **healthcare delivery and health insurance systems**, **employment structure and working conditions**, **and role differentiation between generalists and specialists**. They identified **the need to change the** **traditional work culture** as a prerequisite for adapting the hospitalist model to Japan. Key changes essential for this transition included creating **incentives for work**-**life balance**, advancing **task shifting to other healthcare workers**, and **overcoming cultural barriers and misconceptions**. **Practical strategies for implementation in Japan** require the **integration and reorganization of healthcare resources**, starting from **pilot programs in large hospitals**. To achieve immediate progress, they suggested specific measures such as **establishing structured handoffs and equitable workloads**, enabling **remote access and digital flexibility, and delegating authority and proxy orders**. However, they believed that **public acceptance and readiness** were essential for adopting the new service.
Theory description
•Physicians who choose to become hospitalists in the United States tend to have an affinity for and orientation toward general internal medicine.
•Hospitalists, in their workstyle, do not receive incentives such as procedural fees, but they are satisfied with maintaining a good work-life balance and having shift schedules with clearly defined on and off times.
•Regarding salaries, they consider their compensation reasonable in relation to labor and hourly workload compared with other subspecialty physicians, mainly due to the favorable work-life balance.
•The introduction of the hospitalist system in Japan depends on public demand and requires demonstrating evidence of its necessity in Japan, along with promoting understanding among users receiving care.
•The advantages of Japan’s national health insurance system include good accessibility and relatively low costs; however, from the perspective of healthcare efficiency, it is necessary to strengthen the gatekeeping function of family physicians and reconsider the balance between generalists and specialists.
•Recruitment of hospitalists can begin with the consolidation of medical institutions and the establishment of hospitalist departments within large-scale hospitals.
•To introduce the system, it is necessary to delegate tasks to multiple professionals, including nurses; abolish traditional workstyle practices through cultural change, such as eliminating overtime and holiday work; and transition from an attending physician system to a team-based system.
•Although there may be legal restrictions, it is highly feasible―depending on operational innovation―to authorize remote access to medical records from outside the hospital and to permit order entry following delegation and subsequent approval by other professionals.

### Theme 1. Systemic and structural differences between Japan and the United States

#### Subtheme 1.1. Healthcare delivery and insurance systems

Participants contrasted Japan’s universal health insurance system―valued for its accessibility, low costs, and patient autonomy―with the U.S. system. They viewed insurer-driven regulation in the United States as facilitating standardized and accountable inpatient care, whereas Japan’s model was perceived as historically prioritizing access over efficiency, a key strength for maintaining equity. However, limited gatekeeping and unrestricted specialist access in Japan were identified as barriers to coordinated care. Accordingly, participants emphasized the need for stronger gatekeeping and a more balanced generalist-specialist distribution to sustain universal coverage.

*“In the U.S., the system-based approach standardizes care… because insurance companies won’t pay otherwise.”* (Hospitalist B)

*“Japanese healthcare hasn’t historically focused on efficiency… more efficient resource management will become essential.”* (Hospitalist C)

#### Subtheme 1.2. Role differentiation between generalists and specialists

Participants reported significant role overlap in Japan, where specialists often provide general inpatient management, resulting in role ambiguity and concentration of workload. In contrast, hospitalist practice in the U.S. was characterized by a clear delineation between hospitalists and subspecialists. This division of labor was perceived as enhancing operational efficiency, predictability of workflow, and accountability.

*“Japan traditionally prioritizes specialization, so specialists often perform general internal medicine tasks… In the U.S., specialists focus solely on their field.”* (Hospitalist C)

*“Specialists in the U.S. are rarely employed by hospitals… they often run private clinics and see their inpatients during breaks.”* (Hospitalist C)

#### Subtheme 1.3 Employment structure and working conditions

Participants emphasized shift-based employment as a defining feature of hospital medicine in the U.S. Clearly bounded schedules, separation of day and night shifts, and compensation independent of procedural volume were viewed as promoting manageable workloads and supporting physician well-being. In contrast, Japan’s continuous-duty culture was regarded as both culturally entrenched and structurally incompatible with work style reform.

* “I was attracted to the flexible work style, such as one week on and one week off, which doesn’t exist in Japan.”* (Hospitalist C)

* “We don’t work consecutively, such as a day shift followed by a night shift… contracts usually specify day or night shifts in advance.”* (Hospitalist C)

### Theme 2. The need to change the traditional work culture

#### Subtheme 2.1. Incentives for work-life balance

Participants described shift-based systems as essential to promoting long-term professional sustainability and physician well-being. Although continuity of care in Japan was associated with professional satisfaction, continuous responsibility was linked to cumulative exhaustion. Consequently, scheduled rest and defined off-time were emphasized as crucial for maintaining motivation and a healthy work-life balance.

*“Hospitalists work one week and have the next week off, without being called at night, which allows time for family.”* (Hospitalist A)

*“[In Japan], continuity of care offers high professional satisfaction, but it can lead to exhaustion. Occasionally taking breaks to refresh is important for maintaining work-life balance.”* (Hospitalist B)

*“Most hospitalists seem to maintain their motivation by engaging in hobbies or other activities during their time off.”* (Hospitalist B)

#### Subtheme 2.2 Task shifting to other healthcare professionals

Participants described delegation to nurses and other allied health professionals as a central component of hospital medicine in the U.S. Practices such as nurse-initiated orders, rehabilitation-led discharge planning, and multidisciplinary care were viewed as reducing physician-centered workload and redefining physicians’ roles as coordinators of care. In Japan, by contrast, physicians’ continued responsibility for non-medical tasks was attributed to legal and institutional constraints.

* “In Japan, doctors are tasked with various non-medical duties and even jobs that could be done by others.”* (Hospitalist C)

*“In the United States, Advanced Practice Registered Nurses―similar to specialized nurses―handle nighttime issues and other tasks to support physicians.”* (Hospitalist A)

*“In the United States, rehabilitation staff determine discharge destinations… this shared decision-making involves social workers and case managers, distributing responsibility rather than burdening doctors alone. Physicians act more as coordinators.”* (Hospitalist C)

#### Subtheme 2.3 Overcoming cultural barriers and misconceptions

Participants identified several cultural norms in Japan―such as the perceived obligation to follow patients “until the end,” reluctance to delegate tasks, and acceptance of excessive overtime―as barriers to systemic change. In contrast, acceptance in the U.S. of complete handoffs and protected time off was seen as legitimizing professional boundaries. Consequently, a shift from individual responsibility to team-based care was viewed as essential for long-term sustainability.

*“Japanese culture seems to encourage doctors to follow their patients until the end… there is often guilt in doing otherwise.”* (Hospitalist C)

*“Working without breaks isn’t necessarily in the patient’s best interest… hospitals need to foster a system of shared patient care.”* (Hospitalist C)

### Theme 3. Practical strategies for implementation in Japan

#### Subtheme 3.1 Integration and reorganization of healthcare resources

Participants identified fragmentation of inpatient services and the predominance of small hospitals as major barriers to implementing hospitalist practice. They proposed regional collaboration, consolidation, and hospital mergers as necessary strategies to ensure staffing redundancy and maintain continuity of care.

*“Without centralization, if a single doctor leaves, there’s no one to take over their patients.”* (Hospitalist A)

*“Hospital mergers or the creation of large medical centers may be the only solution.”* (Hospitalist B)

#### Subtheme 3.2 Pilot programs in large hospitals

Participants proposed initiating implementation through pilot programs in large or consolidated hospitals with sufficient staffing, training capacity, and administrative support. Successful early models were viewed as mechanisms for institutional learning and broader diffusion.

*“Larger hospitals can hire more doctors… small hospitals would require double or triple the current staff.”* (Hospitalist B)

*“These systems could start in model hospitals and expanding if successful.”* (Hospitalist B)

#### Subtheme 3.3 Establishing structured handoffs and equitable workload

Participants emphasized structured handoffs, capped shifts, and balanced patient assignments as core mechanisms for promoting fairness and managing workload. Clear separation between inpatient and outpatient roles, as well as between day and night responsibilities, was regarded as a feasible approach to reducing burden and preventing burnout.

*“Patients admitted overnight or during the previous day are handed off. Each hospitalist manages up to five patients, with cases distributed evenly.”* (Hospitalist A).

*“Here, shift times are strictly managed, and patient assignments consider workload.”* (Hospitalist C)

#### Subtheme 3.4 Remote access and digital flexibility

One participant, reflecting on practice within their own service, highlighted remote access to electronic health records and off-site order entry as means of enhancing temporal and spatial flexibility. These features were viewed as improving efficiency without requiring constant hospital presence and as potentially feasible in Japan if supported by appropriate safeguards.

*“We can access medical records and place orders from home.”* (Hospitalist A)

*“I wake up early to review patient records and plan treatments at home. By the time I start rounds, most of the documentation is complete, making the work more efficient.”* (Hospitalist A)

#### Subtheme 3.5 Delegation of authority and proxy orders

Allowing nurses to place provisional orders with subsequent physician co-signature was described as a concrete form of task shifting that could streamline workflows while preserving accountability. One hospitalist further emphasized the importance of establishing systems that permit nurses to place provisional or proxy orders, which physicians later verify or co-sign, to enhance efficiency while maintaining professional responsibility.

*“It might be helpful to authorize nurses to place orders, with doctors later co-signing.”* (Hospitalist A)

### Theme 4. Public acceptance and readiness

Participants agreed that public understanding of the hospitalist role is critical for successful implementation in Japan. They anticipated that adoption would depend on evidence demonstrating improved outcomes, greater efficiency, or reduced costs, accompanied by supportive policy measures. Public demand was therefore conceptualized as a function of demonstrable value and institutional legitimacy.

*“If data emerge and public opinion pushes for change, it might happen.”* (Hospitalist A)

## Discussion

This qualitative analysis suggests that the hospitalist work style aligns closely with the goals of Japan’s work style reform, with the identified themes interacting across systemic, cultural, and operational dimensions. Participants described institutional and structural differences between Japan and the U.S., highlighted cultural norms related to continuity of care, delegation, and overtime, and identified practical strategies―such as structured scheduling, team-based management, and standardized handoffs―that could be adapted within Japan’s existing healthcare framework.

At the operational level, structured, shift-based scheduling and standardized handoffs emerged as practical entry points for implementation. Transparent and predictable schedules―such as a “one week on/one week off” rotation―were viewed as enhancing flexibility, reducing excessive overtime, and improving job satisfaction and work-life balance. In the U.S., hospitalists report higher job satisfaction than general internists despite experiencing comparable levels of burnout ^(11)^, and well-designed handoff protocols are considered essential for maintaining continuity of care ^(12), (13)^. Team-based inpatient management was also emphasized as an effective means of distributing workload while maintaining quality and patient safety. Consistent with prior evidence, hospitalist practice has been associated with greater efficiency, reduced costs, and improved patient outcomes ^(14), (15)^. Given the established relationship between workload and burnout, strengthening work control and ensuring adequate rest are critical. Although the estimated burnout rate among Japanese internists is approximately 30% ^(16)^, persistent challenges―including limited work control, frequent on-call responsibilities, and insufficient time off―continue to undermine physician well-being ^(17), (18)^, underscoring the importance of adopting these operational practices in Japan.

At the system level, several strategies proposed by participants appear feasible within Japan’s existing legal and healthcare frameworks. To enhance inpatient efficiency while maintaining the continuity characteristic of Japanese medical practice, multidisciplinary and interprofessional collaboration was regarded as essential ^(19)^. Japan has made gradual progress in task shifting: medical clerks are now widely employed, although often underutilized ^(20)^, and since 2015, nurses who complete MHLW-approved training have been permitted to perform specified medical procedures ^(21)^. Building on this foundation, certified nurses and nurse practitioners are increasingly expanding their roles; however, their scopes of practice and legal accountability remain incompletely defined ^(22)^. Delegating authority without compromising quality or safety may alleviate physician workload and promote more sustainable work styles. Given that larger institutions generally possess the infrastructure and resources necessary to implement such systems ^(23)^, pilot programs in tertiary hospitals represent a pragmatic entry point for evaluating the hospitalist model. Participants also highlighted legislative and technological innovations―including remote access to electronic health records and post-co-signing systems―as mechanisms to streamline workflows and strengthen interprofessional collaboration. Furthermore, the expansion of online medical practice following coronavirus disease 2019-related reforms may further support flexible work arrangements ^(24)^. From an educational perspective, Japan’s postgraduate core competencies already emphasize team-based care and quality and safety management ^(25)^; integrating hospitalist-oriented practices―such as structured inpatient rotations and formal handoff training―into early residency programs is therefore aligned with national reform priorities.

At the cultural level, participants emphasized that, beyond system design and operational change, the principal barriers to sustainable reform lie within professional culture. Transforming traditional norms requires a fundamental shift from individual responsibility toward shared, team-based care, accompanied by the acceptance of defined boundaries such as structured handoffs and protected time off. Institutions may also need to strengthen support for physicians in balancing personal and professional life and to recognize well-being as a core organizational value ^(26)^. Although the Work Style Reform Bill has contributed to reducing excessive overtime, achieving deeper cultural change toward genuinely balanced labor practices remains challenging ^(27)^. In this context, wellness-centered leadership―placing healthcare workers’ well-being at the heart of organizational performance―is essential for preventing burnout and enhancing professional fulfillment ^(28)^. Such cultural transformation is likewise necessary to address persistent system-level differences between Japan and the U.S., including role differentiation, approaches to delegation, and norms surrounding handoffs and protected time.

Finally, broad implementation will depend on public acceptance and readiness. Demonstrating that hospitalist-led care improves clinical outcomes, patient satisfaction, and resource utilization will be essential for gaining both policy and societal support. Early evidence from Japanese hospitals adopting hospitalist-style systems indicates improvements in workflow efficiency and quality of care ^(29), (30), (31)^. As observed in the U.S., positive effects on patient experience and satisfaction are also likely to strengthen the case for wider adoption ^(32), (33)^.

Several limitations warrant consideration. First, the sample size was small, all participants were male, and all were affiliated with a single U.S. teaching hospital, which may limit the transferability of findings. Second, although participants completed their primary medical education and early clinical training in Japan, their subsequent professional trajectories may reflect self-selection toward international or non-traditional practice patterns. Third, interviews were conducted in 2018, prior to the full enforcement of physician work-hour regulations in Japan in 2024. Although participants’ perspectives captured enduring structural and cultural features, subsequent policy and institutional developments may influence the present relevance of the findings. Accordingly, the results should be regarded as exploratory rather than conclusive.

Despite these limitations, the present findings offer a distinctive set of practical insights. The purposive inclusion of Japan-trained physicians practicing as hospitalists in the U.S. provided a rare cross-cultural perspective that revealed not only systemic and cultural barriers but also work style practices that can be implemented immediately, independent of major legal or institutional reform. In particular, structured scheduling, standardized handoffs, and team-based inpatient care emerged as actionable strategies for improving work control, continuity of care, and physician well-being within Japan’s existing framework.

Overall, the hospitalist work style offers valuable reference points for Japan despite institutional differences between the two countries. This qualitative analysis identified organizational, cultural, and operational practices that may inform the ongoing work style reform. These practices are anticipated to enhance physicians’ quality of life and well-being while maintaining patient safety and quality of care. To expand these approaches beyond isolated institutional initiatives, continued accumulation of evidence―along with systematic case documentation and coordinated reform efforts―will be essential.

## Article Information

### Acknowledgments

We thank Editage (www.editage.jp) for English language editing.

### Author Contributions

Michito Sadohara and Kunihiko Matsui conceived the conception and design of this research. Michito Sadohara conducted data acquisition. Michito Sadohara and Kunihiko Matsui conducted analysis and interpretation of the data. Michito Sadohara wrote the draft of the manuscript. Kunihiko Matsui supervised the methodological design, analysis, and interpretation of the data, and reviewed and wrote the manuscript. Both authors approved the final version for publication.

### Conflicts of Interest

None

### IRB Approval Code and Name of the Institution

This study protocol was considered and approved by the Research Ethics Committee of Kumamoto University (Approval No. 1572).

### Disclaimer

Kunihiko Matsui is one of the Editors of JMA Journal and on the journal’s Editorial Staff. He was not involved in the editorial evaluation or decision to accept this article for publication at all.
